# Solid-state batteries: from ‘all-solid’ to ‘almost-solid’

**DOI:** 10.1093/nsr/nwad098

**Published:** 2023-04-11

**Authors:** Hanyu Huo, Jürgen Janek

**Affiliations:** Institute of Physical Chemistry, Justus Liebig University Giessen, Germany; Center for Materials Research (ZfM), Justus Liebig University Giessen, Germany; Institute of Physical Chemistry, Justus Liebig University Giessen, Germany; Center for Materials Research (ZfM), Justus Liebig University Giessen, Germany

## Abstract

The ‘all-solid’ concept is not necessarily the most rewarding target, and ‘almost-solid’ may rather be the most feasible strategy.

Lithium-ion batteries (LIBs) have been the undisputed leading technology in electrochemical energy storage since they were commercialized in 1991. Since then, the mass manufacturing of LIBs has reached maturity, and we have also seen the realization of high energy density, long cycling stability and low cost. LIB technology enabled the huge success of mobile consumer electronics, with its usage in electric vehicles and other advanced devices. Due to the use of organic solvents for the electrolytes, LIBs are sensitive to high temperatures, lose performance at low temperatures and show inherent safety risks with increasing energy density (Fig. 1a). As the performance of current LIBs is also limited, next-generation battery technologies are being intensively investigated, especially given the ever-increasing demand for high energy density as well as high power density.

**Figure 1.**
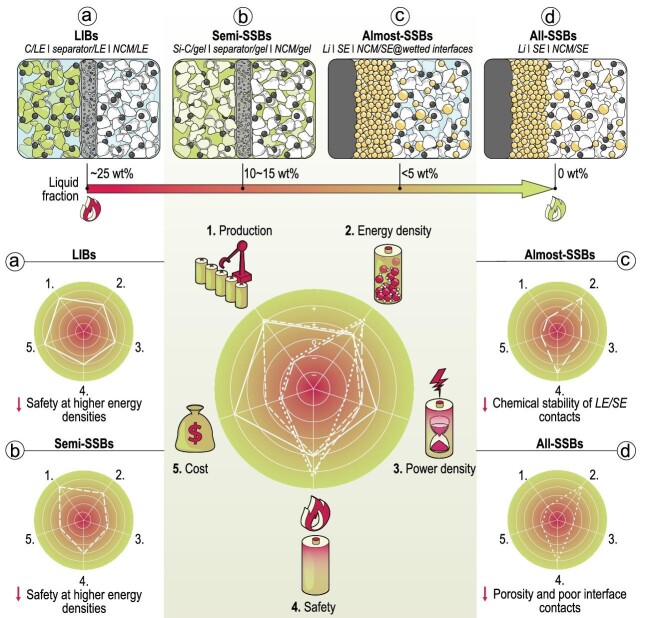
Comparison of various cell concepts with different fractions of liquid components.

All-solid-state batteries (all-SSBs) have emerged in the last decade as an alternative battery strategy, with higher safety and energy density expected [1]. The substitution of flammable liquid electrolytes (LEs) with solid electrolytes (SEs) promises improved safety. Moreover, the possibility of bipolar stacking, and the use of high-voltage cathodes and a lithium metal anode can potentially improve the energy density of SSBs compared to LIBs. The work reported by Kanno and other Japanese scientists, in which an SE (i.e. Li_10_GeP_2_S_12_) showed a lithium ion conductivity higher than LEs [2] for the first time, further fueled interest and confidence in the practical applications of SSBs.

Although many companies announced their all-SSB layout based on different SEs a long time ago, most companies experienced difficulties when it came to launching any all-SSB products. The critical bottleneck comes from the SEs and their properties, especially their interface issues (Fig. 1d) [3]. Oxide SEs made of authentic ceramics not only require high temperature/pressure sintering for densification but also show slow interface kinetics, mainly due to

poor interface contact. The use of sulfide/halide SEs is inevitably affected by moisture and requires dry-room operation, although cold-press densification is feasible for relatively soft sulfide/halide SEs. All-SSBs based on inorganic SEs in general suffer from chemo-mechanical issues (either contact loss, interphase formation, or both) during operation, even if the SE/electrode contact is sufficient after assembly. Polymer-based all-SSBs have the advantage of apparently good SE/electrode contact but require an elevated operation temperature due to the insufficient ionic conductivity of polymers at room temperature. Interface degradation occurs at high potentials considering the narrow electrochemical window of typical polymer SEs. Current all-SSBs are therefore still less competitive than LIBs with regard to cycling stability, rate performance and energy density. Moreover, the high price of SEs, unmatured production lines and additional devices for stack pressure create barriers for the scaling up of all-SSBs.

Recently, hybrid battery concepts have emerged as an intermediate route, where both SEs and LEs are involved in pursuing higher safety and energy density than LIBs while mitigating the chemo-mechanical problems in SSBs (Fig. 1b). These hybrid solid-liquid concepts have been advanced by various scientists and companies, and are often referred to as ‘semi-’, ‘quasi-’ or ‘pseudo-’ SSB concepts—but also often simply considered as SSBs in public, which may be misleading [4]. In the case of polymer-based cells, adding a (substantial amount of) LE leads to the formation of gel polymer electrolytes with improved ionic conductivity; this method is widely used. In the case of inorganic SEs, the LE ideally fills voids and gaps, increases the electrochemically active interface areas, and thus lowers the electrode tortuosity and impedance.

Semi-SSBs share major materials, similar manufacturing processes and similar production lines with current LIBs, thus are easier to scale up compared to all-SSBs. Many companies demonstrated their semi-SSB products successively. Energy densities have been announced to be ∼350 Wh kg^–1^, with claims of up to 400 Wh kg^–1^ achieved by optimized pack structures and alternative electrodes with higher specific capacities. For example, NIO launched a 150 kWh semi-SSB consisting of a hybrid electrolyte, Si-C composite anode and ultra-high nickel cathode, with an energy density of 360 Wh kg^–1^, enabling a 1000 km driving range on a single charge [5]. The fraction of liquids in these semi-SSBs was never revealed, yet it is speculated to be 10 wt% to 15 wt% based on typical gel polymer electrolytes. However, the targeted increase in safety will probably only be achieved if the fraction of added liquid gets much smaller. The liquid fraction should probably be <5 wt% for almost-SSBs (Fig. 1c) [6]. However, even all-SSBs exclusively containing solid components may still have a risk of thermal runaway [7]. Clearly, the safety issues of SSBs will be different from LIBs, yet clear-cut proof still requires in-depth evaluation.

Current semi-SSBs, often considered SSBs for simplicity, are based on the modification of LIBs, where the ion transport in both electrolyte and electrode is mainly governed by an LE. We assume that this modification may be a marketing strategy, rather than a real advantage of SSBs over LIBs, especially if only a small amount of SE is added to what is otherwise an LE. From our point of view, the liquid component should only serve as an interface agent to keep the liquid fraction low, for safety reasons, and the solid/liquid interfaces need to be chemically stable. Otherwise, degradation and interphase formation may occur, leading to a performance decrease in the long term and compromising the whole concept. Since both oxide SEs and sulfide SEs show a strong tendency toward interaction with organic solvents, small-molecular-weight polymers are applied to substitute conventional LEs to stabilize the interfaces [8]. Super-concentrated (or solvent-in-salt) electrolytes or solvate ionic liquids are also suggested, where the strong interaction between Li^+^ ions and electronegative elements in the polar solvents can alleviate the reactivity of solvents, thus stabilizing the liquid/solid interfaces [9,10]. In addition, adding a liquid improves the electrochemical properties of electrodes only if ion transport through the composite is improved and electron transport is not compromised. This requires that ion transfer across the solid/liquid interfaces shows a sufficiently low resistance—which has rarely been proven. Recently, *in situ* polymerization has been used to solidify an originally liquid component in semi-SSBs, thus lowering the liquid content [11]. The liquid acts as a ‘self-healing’ additive at the beginning. Once it polymerizes, it still helps to keep sufficient contact even in the case of volume changes of electrodes.

Thin separators (<60 μm) and thick cathodes (>4 mAh cm^–2^) are required to boost the energy density (>350 Wh kg^–1^) of almost-SSBs [6]. This not only needs advanced fabrication processes with optimized microstructures, but also requires solid/liquid electrolytes with good ionic conductivity to ensure fast ion transport. Organic-inorganic composite SEs, which combine the advantages of both organic and inorganic SEs, show good ductility and mechanical strength, good processability, and sufficient ionic conductivity for mass production. The porosity of cathode composites should be decreased to meet the target of <5 wt% (pore-filling) LE for almost-SSBs. Techniques, such as microstructure optimization and calendering in a dry process with different shear forces, need to be explored to minimize porosity without compromising the structural integrity of cathodes. In addition, we highlight the need for a high yield strength of SEs instead of a high Young's modulus to mitigate the interface stress by the volume change of electrodes [12]. The quantitative study of the role of a liquid component on chemo-mechanical properties deserves further investigation as we transition from all-SSBs to almost-SSBs. Not least, lithium dendrite suppression should be considered once lithium metal is used as the anode.

The ‘all-solid’ concept is not necessarily the most rewarding target; rather, ‘almost-solid’ may be the most feasible strategy. A small fraction of liquid interface additive may lower the electrode impedance, help to mitigate contact loss when there are local cracks and keep long-term stability under the influence of cyclic volume changes of active materials—provided that LE and SE do not react and cause chemical degradation. To move from current semi-SSBs to almost-SSBs, a smaller liquid fraction (probably <5 wt%) is required to achieve safety targets. Both highly conductive SEs and LEs (i.e. >10 mS cm^–1^ at room temperature) and corresponding solid/liquid interface stability need to be achieved and further explored en route. The cost of mass production should be decreased via optimized manufacturing processes and innovative material recycling routes. In any case, we are confident that we will see the commercial success of almost-SSBs in the near future.
